# Optimized expression of alternative oxidase

**DOI:** 10.1038/s41434-022-00340-7

**Published:** 2022-05-17

**Authors:** Jiri Neuzil

**Affiliations:** 1 School of Pharmacy and Medical Science, Griffith University, Southport, QLD Australia; 2 Institute of Biotechnology, Czech Academy of Sciences, Prague-West, Czech Republic

**Keywords:** Biological techniques, Biomarkers

In their paper titled “Alternative oxidase encoded by sequence-optimized and chemically-modified RNA transfected into mammalian cells is catalytically active” Giordano et al. [1] report on a very interesting paradigm that concerns mitochondrial function.

Alternative oxidase (AO) is a highly intriguing protein expressed by lower eukaryotes, such as the invertebrate *Ciona intestinalis*. This enzyme with the size of <40 kDa has the combined activity of mitochondrial respiratory complexes III and IV (CIII and CIV). It accepts electrons from reduced form of coenzyme Q (CoQH_2_) to maintain its redox-cycling without pumping protons across the inner mitochondrial membrane. Given the fact that CIII and CIV comprise the total of 24 subunits, 20 encoded by nuclear DNA and 4 by mitochondrial DNA (mtDNA) while AOX is a single polypeptide with the same function as a recipient of electrons from CoQH_2_, this disparity is one of the many curiosities of evolution.

It has been documented that AOX can compensate for defects in oxidative phosphorylation (OXPHOS) in invertebrates and in vertebrates [2–4], and alleviate them from debilitating dysfunction of highly energetically demanding organs, such as cardiomyopathy [5]. We have found in our research that AOX is sufficient to drive cancer cell proliferation and tumor progression from cancer cells with the absence of OXPHOS due to deletion of mtDNA, linking mitochondrial respiration and de novo pyrimidine synthesis [6]. This paradigm was recently corroborated by others, showing the crucial role of CoQ redox-cycling in tumor progression and the compensatory role for AOX as an electron acceptor [7].

In their paper, Giordano et al. [1] reason that expression of AOX can alleviate conditions that are typified by dysfunctional respiratory function with the caveat that long-term AOX expression may cause persistent “adaptive metabolic re-modeling” that can be ultimately detrimental. They propose that short-term, i.e., transient expression of AOX may be a better strategy to intervene with pathologies by single course of treatment. The authors thus followed the strategy of encoding AOX by chemically modified RNA (cmRNA), where the sequence was optimized for mammalian systems. The new approach was successfully tested using several human and mouse cell lines, including immortalized mouse embryonic fibroblasts, primary mouse lung smooth muscle cells, and human non-small cell lung carcinoma cells.

Furthermore, the authors propose that transfection of cells with cmRNA results in relatively fast expression of the AOX protein, since it requires only translation, while the transcriptional step is not needed. To control for the activity of AOX, they also prepared a mutated (presumably inactive) AOX cmRNA construct. They show that the AOX protein is expressed in the cells as soon as 6 h after transfection and starts to decline after 2 days with its levels close to zero on day 7. The cells exert the AOX activity only in the case of transfection with the active form of the enzyme. Furthermore, there is no effect of AOX on components of mitochondrial respiratory complexes, neither is there any effect on mitochondrial respiration dependent on complex I or complex II. This documents that expression of AOX using the optimized cmRNA construct results in rapid and transient expression of the AOX protein without interfering with the endogenous function of mitochondria.

While these are very nice and inspiring results, it would be good to document that in this way, expression of AOX is also active in cells that lack mtDNA, i.e., in cells that are respiratory incompetent. Notwithstanding this, the approach shown by the authors can be used for alleviating in various pathologies typified by dysfunctional respiratory function. Thus, this publication lays foundation for future interventions. However, it is yet to be seen whether this approach can be utilized as proposed. If so, this will be a great contribution to potential solution of difficult-to-treat conditions.
